# The inefficacy of antiangiogenic therapies

**DOI:** 10.1186/2040-2384-2-27

**Published:** 2010-12-10

**Authors:** Domenico Ribatti

**Affiliations:** 1 Department of Human Anatomy and Histology, University of Bari Medical School, Bari, Italy

## 

In 1971, Judah Folkman published in the "New England Journal of Medicine" a hypothesis that tumor growth is angiogenesis-dependent and that inhibition of angiogenesis could be therapeutic [1]. This article also introduced the term antiangiogenesis to mean the prevention of new vessel sprouts from being recruited by a tumor.

In the last 35 years, it has been estimated that > 200 companies have worked and are still working in the area of angiogenesis and several of the compounds that modulate angiogenesis are currently being evaluated in clinical trials. A list of approved antiangiogenic drugs approved for clinical use is available in the Table 1.

**Table 1:** List of Antiangiogenic Drugs Approved for Clinical Use

Drug	Target	Company	Indication
Avastin (Bevacizumab)	VEGF	Genentech	mCRC, NSCLC, Advanced breast cancer
Erbitux (Cetuximab)	EGFR	Imclone	mCRC & Head and Neck cancer
Vectibix (Panitumumab)	EGFR	Amgen	mCRC
Lucentis (Ranibizumab)	VEGF	Genentech	Wet Age-related macular regeneration
Macugen (Pegaptanib)	VEGF	OSI Pharmaceuticals	Wet Age-related macular regeneration
Endostar (Endostatin)	Angiogenesis inhibitor	Shangdong Simcere Medgen	Lung cancer
Sorafenib (Nexavar)	VEGFR, PDGFR & Raf	Bayer AG/Onyx	Advanced RCC
Sunitinib (Sutent)	VEGFR, PDGFR & c-kit	Pfizer	Advanced RCC & GIST
Thalomid (Thalidomide)	Angiogenesis inhibitor	Celgene Corporation	Multiple Myeloma
Sorafenib (Nexavar)	VEGFR, PDGFR & c-kit	Bayer AG/Onyx	Advanced RCC
Sunitinib (Sutent)	PDGFR & VEGFR	Pfizer	Advanced RCC & GIST
Dasatinib (Sprycel)	Bcr-Abl & Src	Bristol-Myers Squibb	Gleevec-resistant CML or Ph+ ALL
Lapatinib (Tykerb)	EGFR & Her2/neu	GlaxoSmithKline	Advanced metastatic Her2+ breast cancer
Velcade (Bortezomib)	Proteosome inhibitor	Millenium Pharmaceuticals	Multiple myeloma
Tarceva (Erlotinib)	EGFR	Genentech/OSI	Lung cancer

Even if the majority of pre-clinical studies have shown that the growth of all experimental tumors can be effectively inhibited by various antiangiogenic agents, the clinical benefits of antiangiogenic treatments are relatively modest, and in the majority of cases, the drugs merely slow down tumor progression and prolong survival by only a few more months.

The most promising antiangiogenic agents that are in clinical development at this moment include bevacizumab, the humanized anti-monoclonal antibody anti-VEGF approved for use in combination with cytotoxic agents [2], as well as small molecules receptor tyrosine kinase inhibitors (RTKIs), approved as single agents, and including sunitinib, an oral inhibitor of VEGFR-2, PDGFR, FlLT-3, and c-KIT, and sorafenib, an inhibitor of the Faf/MEK/Erk and the VEGFR and PDGFR signaling pathways. These agents are generally well tolerated, but the treatments may be accompained by distinct adverse effects, including hypertension and proteinuria.

In a communication in the 2003 ASCO Meeting, Hurwitz and co-workers reported that bevacizumab/IFL (irinotecan/fluorouracil/leucovirin) combination led to a significantly prolonged survival and had a better ability to shrink tumors that IFL alone. These results led the FDA to approve the use of bevacizumab in patients with metastatic colorectal cancer and Hurwitz and co-workers have published the results of this study in 2004 [3]. In December 2005, sorafenib received FDA approval for the treatment of renal cell carcinoma [4] while sunitinib received FDA approval in January 2006 for patients with gastrointestinal stromal tumors (GIST) and advanced kidney cancer [5,6].

Clinical studies have shown benefits in relapsed-free survival for metastatic colorectal cancer, advanced non-small cell lung cancer, renal cell carcinoma, hepatocellular carcinoma, metastatic breast cancer, GIST and in glioblastoma [7,8], but overall survival benefit has not yet been seen [9], with the exception of bevacizumab treatment in renal cell carcinoma as a single agent [10], or in metastatic breast cancer in combination with a taxane chemotherapy [11].

The most impressive clinical response occurred in the low dose bevacizumab plus chemotherapy with a statistically significant median overall survival (21.5 months) versus fluorouracil/leucovorin alone (13.9 months) or high-dose bevacizumab plus fluorouracil/leuocovirin (16.1 months) [12].

Autocrine VEGF signaling to promote malignant cell survival is also a common feature in haematological malignancies, suggesting that anti-VEGF/VEGFR targeted therapy would promote direct killing of tumor cells, as well as inhibit angiogenesis. VEGF-directed therapy has been investigated also in hematological malignancies, most commonly in acute myeloid leukemia, myelodysplastic syndrome, and in non-Hodgkin lymphoma.

Clinical trials involving anti-VEGF agents induce only a modest improvement in overall survival, measurable in weeks to just a few months, and various tumors respond differently in human patients to these agents.

These two principal findings could depend by different synergistic causes:

1) Lack of understanding of which patients will show the benefit of these agents and occurrence of drug resistance [9,13,14]. This is due to the absence of reliable surrogate markers of angiogenesis and antiangiogenesis to demonstrate the efficacy of antiangiogenic agents in clinical trials and for the monitoring of these agents [15].

2) Endothelial cells isolated from various tumors acquired genotype alterations, exhibiting aneuploidy, abnormal multiple chromosomes, and aberrant chromosomal architecture [16]. It has been proposed that proximity of tumor cells and endothelial cells within the tumor microenvironment may be responsible for the genotype alterations [17]. Genetic alteration of endothelial cells leads to altered antiangiogenic targets and resistance.

3) Antiangiogenic therapies may sometimes promote invasion and metastasis [18]. It has been demonstrated that sunitinib, a multi-targeted receptor tyrosine kinase inhibitor of VEGF and platelet derived growth factor (PDGF) signaling and the anti-VEGFR-2 antibody DC101 stimulated the invasive behavior of tumor cells despite their inhibition of primary tumor growth and increased overall survival in some cases [19,20].

4) Inherent or acquired resistance to anti-VEGF drugs can occur in patients, leading in some cases to a lack of response and in others to disease recurrence, although discontinuation of the therapy at the time of progression is a factor limiting the effectiveness of antiangiogenic therapy [21]. In the meantime, prolonged VEGF leads to vascular pruning and endothelial cell apoptosis, release of cytokines by host cells, which may promote tumor re-growth.

5) In most tumors, the vasculature is altered showing increased permeability, vessel dilatation, decreased/abnormal pericyte coverage and abnormal basement membrane structure. While VEGF neutralization can initially limit tumor proliferation due to its antiangiogenic effect, it can also result in transient vascular normalization with improved oxygenation and perfusion [22], favouring drug delivery. However, in gliomas normalization of the vascular bed involves restoration of the blood-brain barrier, thereby hampering, instead of enhancing, the delivery of therapeutic compounds to tumor cells [23].

6) Prolonged VEGF inhibition increases local hypoxia leading to systemic secretion of other angiogenic cytokines, such as FGF-2 and SDF-1α, which may promote cancer re-growth and metastasis [24]. An analysis of human breast cancer biopsies revealed that late-stage breast cancers expressed several angiogenic cytokines in contrast to earlier stage lesions, which preferentially expressed VEGF [25].

VEGF inhibitors are effective in the antiangiogenic treatment, but genetic mutations, vascular changes, up-regulation of other pro-angiogenic cytokines, promotion of invasion and metastasis, reduce their effectiveness. Further clinical investigations are needed to optimize antiangiogenic treatments in solid and hematological tumors management, as well as the identification of reliable markers that predict the relapse and the response to these therapies.

## Competing interests

The Author declares that he has no competing interests.
